# Comparing adult education systems: Canada and Aotearoa New Zealand

**DOI:** 10.1007/s40955-020-00158-z

**Published:** 2020-05-28

**Authors:** Judith Walker

**Affiliations:** University of British Columbia, Vancouver, Canada

**Keywords:** Canada, New Zealand, Inclusive liberalism, Neoliberalism, Kanada, Neuseeland, Inklusiver Liberalismus, Neoliberalismus

## Abstract

This article examines recent policy initiatives in adult education and training in Canada and Aotearoa New Zealand in relation to political and educational reforms enacted over the previous decades. The paper attempts to deepen our understanding of adult education systems—or lack thereof—in each place, and of neoliberalism, and responses to it.

## Abstract (de)

Dieser Beitrag stellt aktuelle politische Initiativen im Bereich Erwachsenenbildung und Training in Kanada und Aotearoa Neuseeland sowie deren Bezug zu den (politischen) Bildungsreformen der letzten Jahrzehnte vor. Mit diesem Beitrag soll das Verständnis von Erwachsenenbildungssystemen mitsamt ihren Problemen vertieft werden, unabhängig von ihrer Verortung, jedoch in Bezug und Stellungnahme zum Neoliberalismus.

Canada and Aotearoa New Zealand (NZ)^1^ are two well-established liberal democracies with high standards of living, high levels of education, and long histories in nonformal adult education. At the beginning of the second decade of this millennium, both countries are investing in the skill development of citizens in the face of growing automation, general job displacement, contractualisation, increasing immigration and cultural diversity, and a need to address the legacy of colonisation. The reforms in the two countries share many similarities with their emphasis on, e.g., the measurement and alignment of skills, education for the labour market, training marginalised Indigenous populations. Yet, there is a dearth of adult education infrastructure in Canada as well as insufficient coordination across the institutions that do exist. A fragmented fiscal federalism (Krelove et al. 1997), the closure of national adult education organisations and termination of initiatives, and a general lack of cross-sectional, substantive reform have meant there is no such thing as a Canadian adult education ‘system.’ In contrast, New Zealand’s centralised policy making, extreme neoliberal reforms of the 1980s and 1990s and policy continuation of them and reaction to them during the following decades, resulted in the creation of structures and institutions that allowed for a highly coordinated, regulated, professionalised, and centralised adult education system.

This paper examines both countries’ commitments to skill development undertaken by centre-left governments over the past few years, in consideration of the political-economic reforms of the previous decades which have led us to where we are today.

## Why focus on Canada and NZ?

Canada and NZ are interesting to compare given their general political, historical, economical, cultural, and educational similarities, and major differences in the formation of adult education ‘systems.’

First, NZ and Canada are both settler-colonial nations where the British came in (or the French, in the case of Quebec) and took over rather forcibly from the Indigenous peoples. In recent years, major efforts have started in earnest to reckon with the countries’ racist, paternalistic, and colonial histories and legacies (Miller 2017; Sullivan 2016). Canada’s Truth and Reconciliation Calls to Action (TRC 2015) focus on the necessary economic, social, political and educational responses and reparations in the aftermath of church/state run residential schools, in operation from the 1830s until 1996, which were embarrassing hotbeds of cultural, sexual, physical and educational abuse with ensuing intergenerational effects. Aotearoa New Zealand, founded in 1840 on the principle of biculturalism through the signing of the Treaty of Waitangi between Māori and the Crown, is held up as an exemplar in the revitalisation of Māori culture and language, growing political power of Māori, and recognition of sovereignty and land title (Sullivan 2016). The Treaty, and commitments to it, inform educational policy at all levels. However, continuing inequality in educational, social, and economic outcomes between Māori and Pākehā^2^ (see Lloyd 2018; Sutherland 2019) sheds a painful spotlight on the entrenched issues facing colonial nation-states.

Second, there are demographical similarities, albeit with some important differences. Around 70% of the population is Caucasian, and 15% vs 17% Asian (NZ and Canada respectively). However, 16.5% of NZ’s population identified as Māori in the last census with only about 5% of Canadians reporting Indigenous ethnicity. Further, 9% of New Zealanders are of Pacific Island descent (generally referred to as Pasifika) (all statistics taken from Stats Canada 2016; Stats 2019). At the population level, both Māori and Pasifika peoples have lower socioeconomic and educational outcomes (Lloyd 2018; Ministry of Education 2019), as do Indigenous Canadians (Statistics Canada 2016). Canada and NZ also have high immigration rates.^3^

Third, in an analysis of the varieties of capitalism (Esping-Andersen 1996; Soskice and Hall 2001), it is generally accepted that both NZ and Canada are Liberal Market Economies (LMEs) (Soskice and Hall 2001), as opposed to Coordinated Market Economies (CMEs) (such as Germany, Sweden etc.), and liberal welfare states (Esping-Andersen 1996), in contrast to social democratic welfare states (such as Sweden). Compared to many CMEs, (though less so than the US), both countries prize liberal individualism, have weaker unions, have greater competition between firms, tend towards means-tested benefits instead of universal provision (to varying extents), and have higher levels of inequality.^4^ Recent research on adult education systems and political economy, has tended towards examining collective skill formation regimes of certain CMES, such as Germany (see, e.g., Busemeyer and Trampusch 2012). Focusing on NZ and Canada helps build our understanding of what kinds of adult education systems can and do emerge in LMEs in consideration of the broader political economic context.

At the same time, there are many differences between the two countries: one is small (NZ: ~268,000 km^2^), one is very big (Canada: ~10 million km^2^). Larger countries, like Canada, tend towards federalism (e.g., U.S., Australia, Germany, Argentina etc.). In Canada, fiscal federalism means that for the most part the provinces and territories have a large amount of freedom in how they run their social welfare systems, receiving bulk funding from the federal government to do so (Krelove et al. 1997). Canada is the only country in the OECD without a national body of education with adult education organised at a provincial level.

Further, as LMEs or liberal welfare states, the two countries have experienced quite different political economic trajectories over recent decades. Perhaps more than any other economically advanced nation, NZ experienced radical political, economic, and social change starting the 1980s. It moved away from an exceptionally protected welfare system to a radical neoliberal experiment, and later to a more inclusive liberal model (Craig and Porter 2006), which involved reinserting social cohesion, civic participation, and democracy into politics. Its extreme free-market reforms of the 1980s and 1990s became termed “The New Zealand experiment” (Gray 1998). These reforms were partially enabled by the small geography and population of the country (~3 million in the 1980s, ~4.7 million today), as well as due to many other factors, which I explore in more depth elsewhere (Walker 2011). While Canada experienced its fair share of neoliberal reforms, starting with Prime Minister Brian Mulroney in the 1980s and particularly under PM Stephen Harper in the 2000s, its extent of reforms was arguably never as great, nor were policies ever instituted as fast, as in NZ.

## A note on methodology

Some comparative education scholars have cautioned against methodological nationalism and comparing nation-state to nation-state given the importance of both transnational organisations like the OECD and of globalising forces (see, e.g., Robertson and Dale 2008). I would argue that in more recent years, however, we are being brought back to the importance of nation-states and their responses, especially, to global crises, such as the movement of people, trade, and now the Covid-19 pandemic. While there are always problems with national comparisons—especially here in comparing a small centralised state with a federated one—such comparison of systems and of recent policies provides us with insight into how two different LMEs prioritise and approach adult education and training through policy frameworks. In drawing on some categorisations from the field of comparative education, we can describe my approach here as sociological, rather than epistemological, and concerned with the broader geopolitical context and how national governments respond to it (see Manzon 2018). In keeping with the tradition of comparative education research, too, I am attempting to examine the phenomenon of adult education and training systems in each country in relation to their unique socio-cultural-historical-political contexts (Schriewer 2014). It may be of interest and importance to the reader for me to address the question of positionality. I am a New Zealander who has been living in Canada for 16 years and who has been researching adult education in both countries for almost two decades. These are the two worlds I have mostly inhabited and I seek to make sense of both from insider and outsider perspectives. Overall, this study contributes to the relative dearth of comparative adult education research of policy in the broader field of comparative education.

## Understanding neo and inclusive liberalism

Neoliberalism has come to refer to a plethora of phenomena. Here we understand it as a historical movement, an ideology, and a set of policy approaches. What we refer to as neoliberalism began as a movement, beginning in the 1970s and 1980s, as the disillusionment with, and subsequent abandoning of, Keynesianism in the face of stagflation (i.e., concurrent inflation and unemployment, which was counterindicated by the theory. See Murad 1962). This was associated with the growing acceptance of monetarism and supply-side economics (Friedman 1962), which emphasised lowering barriers to production, cutting government spending, and reducing government’s normative goal to growing the economy by getting out the way. In reality, governments began to actively intervene to promote a market society (MacEwan, 1999), with policies underpinned by what I am terming an *ideology of distrust, *for example, of:

- **The public sector **for its supposed inefficiencies and inabilities to generate profits, resulting in increasing privatisation and New Public Management reforms to make the public sector more like a business.
- **The non-profit, volunteer sector **for its purported lack of quality, resulting in professionalisation and privatisation
- **Unions** for interfering in the market and preventing competition, resulting in de-unionisation.
- **Institutions, and the individuals in them**, to be honest, capable, hardworking, and productive, resulting in increased auditing, demands for accountability, quality control, performance indicators, and assessment tools.
- **Qualitative indicators **of quality, resulting in increasing metricisation and quantification.
- **Education for non-labour market purposes,** given the primacy of the market, resulting in an ignoring of community adult education

Craig and Porter (2006) used the term “inclusive liberalism” to describe the reforms undertaken in the 1990s and early 2000s by the World Bank, NZ, and certain other countries, which were a response to the misery wrought from structural adjustment programmes and neoliberalism’s harsher aspects. Governments, such as NZ’s, sought to partially re-embed the economy, in a limited resubordination of the economy to civil society (Polanyi 1957), while focusing on the inclusion of disadvantaged others. Under inclusive liberalism, there is a special focus on seeking to include certain populations (such as Indigenous peoples) in the market who clearly have not benefitted under neoliberal global capitalism. There are many similarities with Giddens’ (1999) Third Way program which carves a middle path between social democratic welfare capitalism and extreme neoliberalism. Importantly, in Gidden’s model, skills and training are endowed with a special role in addressing central social welfare policy concerns and are generally funded accordingly. However, inclusive liberalism or the Third Way can be understood more as a shift in degree not in kind to neoliberalism (Craig and Porter 2006; Gibb and Walker 2011). Certain ideologies and practices remain. It is my contention that both NZ and Canada recently and currently reflect certain inclusive liberal sensibilities. However, Canada never underwent the extent of the more pure neoliberal reforms of NZ.

## Getting the skills we need: Canadian and NZ adult education systems in 2020

Both NZ and Canada have introduced recent policy initiatives to help support adult education and address some of the challenges the countries face, especially with regards to skills shortages and technological disruptions to the labour market. In 2019, both countries announced major skills initiatives: Canada, the *Future Skills Program, *and NZ, the creation of the *New Zealand Institute for Skills and Technology (NZIST). *Yet, while the problems plaguing the two countries share many similarities, the adult education ‘systems’ bear little resemblance to one another. In what follows, I present a general overview of adult education in each place and highlight some of the more recent policy directions.

### Aotearoa New Zealand

In NZ, education is highly centralised and regulated. All post-compulsory education, from adult and community education to universities, falls under the jurisdiction of the Tertiary Education Commission (TEC). In fact, adult, community, and vocational education comprise a significant part of TEC’s mandate and concerns. Governing all education is the New Zealand Qualifications Authority (NZQA) which oversees accreditation and quality control, and enables organisations and educational providers to confer certain credentials and apply for particular pools of funding. The NZQA conducts assessment; liaises with overseas certifying bodies; oversees entrance to university; grants approval of courses; represents institutional authority; and provides accreditation of new courses. Other government departments important to adult education include the Ministry of Education (though much less so), and the Ministry of Business, Administration, and Employment, which is a catch all department that oversees New Zealand Immigration, Employment New Zealand (formerly the Department of Labour), and Housing, Science, Innovation, Urban development, Māori economic development and a host of other functions (see also Walker 2011).

Recent adult education policy initiatives have focused on the vocational and skills sectors and on creating greater unification. The world’s first “Wellbeing budget” (NZ Treasury 2019) in 2019 allotted around $200 million for vocational and trade training programmes. The largest piece of policy news has been the proposed development of NZIST, which will ultimately become NZ’s largest provider of tertiary education, first bringing together the country’s 16 polytechnics and institutes, and then supporting online, workplace and other vocational education in a unified system (TEC 2020). Other reforms underway are the creation of work development councils in the place of Industry Training Organisations (ITOs) and of Centres of vocational excellence. The focus, above all, is on initiatives that allow the country to meet its Treaty obligations and commitments to Māori through education.^5^

The government has also been apportioning greater funds towards workplace literacy and numeracy, the training of adult educators, adult and community education in schools, among other initiatives (TEC 2019a, b). There has been development of the ACE (Adult and Community Education) sector through an emphasis on Adult Literacy, Numeracy, and Cultural Capability (https://ako.ac.nz/alnacc/), and a commitment to embedding literacy in all forms of education (Walker 2011). Assessment of learning has been a crucial part of this, with the development in recent years of a Literacy and Numeracy Adult Assessment tool, containing thousands of questions (which are often culturally specific to NZ), used to measure progress at the cohort level. As the TEC makes clear, usage of the assessment tool is “… a funding condition for [Tertiary Education Organisations] TEOs receiving foundation-level education funding from us.” (TEC 2016).

Although the inclusion of immigrants is a policy concern of the government, it is interesting that NZ (unlike Canada) not only does not offer free English classes to new immigrants, but also stipulates that immigrant applicants “must pay [the NZ government] for ESOL tuition as part of the application”, if their English scores are deemed insufficient. This translates to anywhere from $1700 NZD to $7000 NZD depending on one’s score on an approved English language test. TEC organises the tuition and those accepted as residents have up to five years to complete the classes (NZ Immigration, nd).

The government currently offers one year of free tuition to anyone pursuing post-secondary education and training (including for those who have previously accrued under a year of tertiary education), with plans to provide three free years of tertiary education by 2024 (Labour NZ 2020). Further, the level of proclaimed of consultation in policy creation is quite striking: the TEC asked for input (to be submitted by October 2019) from across the country on the “long-term vision, objectives and actions for the education system”, requesting specific feedback on the draft priorities for the National Education and Learning Priorities (NELP) and the Tertiary Education Strategy (TES) (TEC 2019b). At the time of writing this paper, the government was also in the midst of a massive consultation process to decide upon a final name for the Institute of Skills and Technology (NZ Ministry of Education, 2020).

### Canada

At the federal level, Employment Skills and Development Canada (ESDC) takes responsibility for funding many adult-education related programming through its Office of Literacy and Essential Skills (OLES); Citizenship and Immigration Canada (CIC) also funds many language and settlement services for immigrants and refugees. Organisations offering adult basic education and language programming generally receive both federal and provincial funds, often in the form of time-limited grants. The federal tri-agency research funding body, especially through its Social Sciences and Humanities Research Council (SSHRC), helps steer academic research in adult education in deciding which projects receive financial support. Mapping the terrain of adult education policy in Canada is incredibly challenging, and sharing of programmatic and policy information among provinces, while facilitated through the Council of Ministers in Education (CMEC), is still difficult (see also Elfert and Walker 2020).

Like NZ, Canada has also focused its attention on vocational training and skills development. Established in 2016, the government’s Advisory Council on Economic Growth (2017), proposed the formation of what became the Future Skills program, and recommended in 2017 “an additional $15 billion in annual investments in adult skills development” (p. 1) which would include the establishment of a federally-governed Canada Lifelong Learning Fund. The Future Skills program was established in 2019, comprising a Future Skills Centre and Future Skills Council. The Centre (FSC-CCF) is an independent research centre, collectively run by a university, and two major think tanks, will “develop, test, and measure new approaches to skill development and assessment,” (Government of Canada 2019) apportioning 50% of its funding to “disadvantaged and under-represented groups.” By the beginning of 2020, the Skills Centre had funded six inaugural research projects and recently closed a call for proposals for researching Support for Mid-Career Workers (see https://fsc-ccf.ca/innovation-projects/). The Future Skills Council, comprised of a diverse representation of “technical and subject matter experts from the public, private and not-for-profit sectors,” (Government of Canada) advises the Minister of Employment, Workforce Development and Labour “on national and regional skills development and training priorities” in response to the changing economy (Government of Canada 2019).

Other new policies related to skills include the new Workforce Development Agreements (WDA) with all the provincial and territorial governments, which will provide a total of $5.2 billion over six years (from 2017–2023) to develop and deliver “programs and services that help Canadians get training, develop their skills, and gain work experience” (cited in Walker, forthcoming), and Skills Boost to offer targeted funding for the unemployed who want to “return to school and upgrade their skills.”^6^ The country also undertook a review of “all the programs that relate to skills in order to maximize effectiveness” (cited in Walker, forthcoming), which comprise 106 programs across 30 departments and agencies, resulting in greater commitments made to improve gender and diversity in skills programs.

Other recent federal budget commitments include the establishment and financial support of a Sustainability Development Goals (SDG) unit through Statistics Canada to monitor and report activities (Government of Canada 2018a). Though it appears that the government commitment is more to measuring and reporting rather than to funding actual adult education programming and to (re)building a robust infrastructure.

A new policy directed to the education of immigrants includes the allotment of $400 million over five years to support the *Action Plan for Official Languages 2018–2023,* which focuses on raising language and literacy levels of visible minority newcomer women, and supporting community organisations build their capacity to pursue government contracts or maximize available funding opportunities (Government of Canada 2018b). Initiatives aimed at reducing the educational gap between Indigenous and non-Indigenous Canadians include over $1.5 million to the Native Education College for the Essential Skills for Aboriginal Business project (Hayes 2018a), and further investment into The Indigenous Skills and Employment Training Program to support better paying jobs, actively considering barriers faced by women and further aligning training with community needs (Hayes 2018b).

The government continues to institute the Testing of Workplace Essential Skills (similar to assessment tools in NZ), a framework which drew on an essential skills framework that was first proposed in the 1970s (Shohet and Coutant 2019). In 2018, Futureworx (2019) was funded by OLES to “explore the need for and how best to develop a pan-Canadian soft skills framework.” In the same year, CMEC endorsed six pan-Canadian global competencies to be fostered across all 13 provinces and territories.

We see many similarities in Canada’s and New Zealand’s concern for supporting adult education: a focus on skills building, concern for closing the gaps between Indigenous and non-Indigenous peoples, measurement of competencies, a continuing focus on adult literacy. However, NZ is a centralised, highly regulated adult education system whereas Canada has a plethora of policies at both provincial and federal levels; and though there is a desire for greater collaboration/coordination, there is nothing that really comes close to an adult education system.

## Looking backwards to understand the present

To make better sense of the present, and perhaps even to think into the future, we need to examine recent political economic history of both places in the creation (and, at times destruction), of adult education systems and institutions.

### Canada: A brief history in relation to adult education

In the 1970s and early 80s, Canada was generally a welfare state and under the leadership of the (first) Liberal Trudeau government, in power from 1968 until 1984 (excepting 1979). This time oversaw the declaration of Canada as a multicultural country (through the development of official multicultural policy in 1971), and the proclamation of the Canadian Charter of Rights and Freedoms (aka the Canadian constitution, in 1982). Canada started to embrace free-trade in the 1980s and 1990s, with the signing of CUFTA and then NAFTA. While neoliberal ideologies and New Public Management philosophies started to creep into public policy over the 1990s, it was only under the previous centre-right Conservative government (from 2006–2015) that the country began to experience more forceful and targeted cuts to social spending and deregulation. Canada remained much more of a welfare state over the 1980s and 1990s with a Conservative, then Liberal, government that, to varying extents, championed and supported adult education (Elfert and Walker 2020).

Policies had already been developed in the 1960s to support vocational training, and short-term retraining for unemployed and underemployed workers. Over the 1970s a database was developed at the (then) Department of Manpower and Immigration on Generic Skills for Occupations, that would then go on to form Canada’s framework for essential skills, which included academic reasoning, interpersonal, and manipulation skills (Shohet and Coutant 2019). The Movement of Canadian Literacy was formed which subsequently developed a coalition of 10 literacy organisations throughout the country. It was in 1987 that centre-right Progressive Conservative Prime Minister Brian Mulroney (in power from 1984–1993) committed to the creation of the National Literacy Secretariat (NLS), which then formed part of his re-election platform. The NLS was entrusted with working with various sectors and organisations nationwide to “ensure Canadians had access to the required literacy skills” (Hayes 2013, p. 4). Thanks to the NLS and other efforts, four additional national adult literacy organisations were formed, and by 1991 there were six such organisations across the country, supporting provision, promotion, and research on adult education. Not only was Canada influenced by the results of the OECD’s first International Adult Literacy Survey (IALS) in the mid-1990s, but was one of the instrument’s main architects which drew on previous surveys of Canadian literacy and on discussions on generic skills that had been taking place since the 1970s (Elfert and Walker 2020). Under the next Liberal government (in power from 1993–2005), the literacy organisations and NLS continued to operate (not without struggles); the Canadian Council on Learning (CCL) was created in 2002 to work with all sectors and organisations of Canadian society to help create “the skills and learning architecture that Canada needs;” and, in 2003, the government committed to working collaboratively to build a pan-Canadian strategy in adult literacy (Elfert and Walker 2020).

Within three months of taking power, the Conservative Harper government (from 2006–2015) folded the NLS into the then Human Resources and Social Development Canada (now ESDC), and by 2007 shut down the NLS. In 2010, the CCL was also defunded, and, due to major cuts in 2014, by 2015 all but two of the six major national literacy organizations had closed their doors (Elfert and Walker 2020). We see new initiatives, as the ones explored above, since the election of the Liberal government yet no real infrastructure. The essential skills framework continues to be hugely influential in training projects, and thinking into the skills needed by Canadian society (Shohet and Coutant 2019).

### NZ: A brief history in relation to adult education

In the 1970s, NZ was largely a closed society with preferential immigration schemes for British immigrants and a highly regulated economy (Cheyne et al. 2005). It was also one of the world leaders in welfare reform (as was Canada, in its pension plan and medicare system, for example). By the 1980s, the country was experiencing numerous economic and social problems. It had a relatively undiversified economy, isolationist and protectionist trade policies, unsustainable levels of agricultural subsidies, and (according to some sources) cripplingly powerful unions (Cheyne, O’Brien, & Belgrave). In the early 1980s, faced with these challenges and in the aftermath of the oil crisis, centre-right National party Prime Minister Robert Muldoon borrowed millions of dollars to build large-scale industrial projects under his “Think Big” initiatives aimed at reducing New Zealand’s reliance on imports. These investments were widely unpopular and expensive, and arguably did little to aid the economy.

Faced with this troubled political and financial situation, the incoming centre-left Labour government, elected in 1984, sought to stimulate the economy under the guidance of Finance Minister Roger Douglas. His economic policies became known as “Rogernomics,” involving radical deregulation, restructuring, decentralisation, privatisation and deunionisation. The “New Zealand experiment” (Gray 1998) was spearheaded by the traditionally leftist Labour government during the mid-to-late 1980s and strengthened and continued under a new-right National party throughout the 1990s. While implementing a policy of market liberalism, the Labour government of the 1980s paid some attention to social equity and concern for the principles of the Treaty of Waitangi; this negotiation over competing ideologies was generally quashed during the 1990s under the more right-wing National party (Walker 2011). The fifth Labour government came to power in 1999, under the leadership of Helen Clark, determined to undo the damage associated with the major policy reforms of the previous era, in a Third Way or Inclusive Liberal approach (Craig and Porter 2006; Giddens 1999).

There was little focus on adult education by the National government in the 1990s, save a few documents in the late 1990s which expressed some concern for lifelong learning and referenced OECD policy concerns and discourses (perhaps as an initial response to IALS results) (Roberts 2000). It is important to examine, however, the changes made to education more broadly through the establishment of the NZQA, which was conferred by the Education Amendment Act of 1990 with the ability to invigilate over most matters educational. While its initial mandate mainly concerned the educational assessment of compulsory schooling, by the late 1990s polytechnics and institutes were brought under its umbrella as the institution was called to offer further quality validation services and more “rigorous assessment”, to exercise greater quality control, and to demand further institutional accountability. It also began to establish policies and criteria related to quality assurance to providers of adult and community education (ACE) (Walker 2011). Private Training Establishments were established throughout the decade, thanks to deregulation, growing to almost a thousand by the end of the decade; they too were provided with the ability to apply for registration under the NZQA (Walker 2011). As Roberts (1997) noted, the NZQA’s rational “scientific” quality allowed it to assume the status of “official knowledge” on educational standards, thus enabling the organisation to radically transform education. It helped bring about a more centralised, cohesive, and seamless education system across all levels.

The following decade saw a further consolidation of power for the NZQA which happened in tandem with the creation of a new ministry, the TEC, established as a result of the Tertiary Education Advisory Commission (TEAC) and consequent Tertiary Reform Bill (2002). The commission and bill set forth a vision of developing tertiary education in New Zealand, and streamlining and incorporating it under one umbrella. Adult education started to receive a lot more focus in the beginning of the 21st Century with the election of the Labour government. An Adult Literacy Strategy (2001) was launched, which connected to NZQA standards; and, the field became further professionalised, with the creation of required certification for adult literacy and vocational training instructors. The NZQA was first directly connected to the Tertiary Education Strategy in 2003, which gave the qualifications authority jurisdiction over quality in all tertiary institutions. Adult and lifelong education was enveloped into the TEC and it is from TEC that many of the main strategies and reforms affecting adult literacy emerged. Now all tertiary education (whether university or basic literacy) provision must meet most of the same policy priorities, abide by the same accountability regimes, and operate on similar funding formulae as other actors in the sector.

Both the NZQA and the Tertiary Education Commission represent the ultimate in centralisation, coordination, and regulation by including everything to do with non-compulsory schooling under one banner. As Zepke (2009) noted, instead of reversing the trends of the previous decades of quality assurance and accountability, there was, and continues to be, a pronounced increase in the focus on continuous improvement in quality.

## Discussion & conclusions: the two adult education ‘systems’

While exceptions exist at provincial levels (especially Quebec), Canada has no real adult education system and no cohesive policy infrastructure. The two main influencers in adult education are arguably think-tanks (as Stone 1996, observed more generally), particularly the Conference Board of Canada which helps to run the newly created Future Skills Centre. There is still an acknowledgment of the importance of a pan-Canadian policy response to adult education, yet “there continues to be 13 jurisdictions shaping their own approach to the field, with few shared tools or methods.” (St-Clair 2016, p. 238). As I have argued elsewhere (Elfert and Walker 2020), the federal system and lack of communication and coordination across provinces is partially to blame here. This prevented the type of centralisation and regulation that took place in NZ. Further, and equally importantly, adult education was never as holistically championed nor infused into the ministries and institutions. There was no comparable neoliberal experiment which could either be capitalised on or responded to; both Conservative and Liberal governments of the 1980s and 1990s invested in adult education. Support for adult education and literacy has waxed and waned, with no real continuity of support—notwithstanding more recent investments by the current Liberal government after the general neglect of the field under the previous Conservative leadership.

In contrast, New Zealand underwent radical changes to its political economy. Under the neoliberal reforms of the 1980s and 1990s, government regulations weakened and the private sector grew. At the same time, governance and regulation increased as power was reconcentrated in the state. Skills training, and adult education more generally, sat in tension with the neoliberal ideology of viewing non-compulsory education as a private good that should, thus, be privately funded. Under Third Way and inclusive liberal ideology, however, skills and adult education became a primary social policy and both a palatable intervention into correcting market anomalies as well as response to the negative effects of global capitalism and technological disruption. What is more, in NZ, the educational structures and systems created throughout the 1980s and 1990s were not only ***not*** undone but furthered in a growing emphasis on skills and workplace adult education, with adult and community education taken also into the fold of the two monolithic entities of the NZQA and TEC.

We also see a neoliberal and continuing inclusive liberal ideology in both places: a concern with a measurement of skills, focus on education for the market place, certain accountability requirements, and attempts to integrate Indigenous peoples into the labour market and to address inequities in participation.

### Pandemic reflections: NZ’s ability to effect rapid, coordinated, and far-reaching reforms

I am editing this paper on the eve that Aotearoa New Zealand has locked down the country for at least a month in the face of the global Covid-19 pandemic. At the first signs of community transmission, the country provided less than 48-hours notice in ordering everyone to work from home and cease movement within the country (after having closed the external borders days ago), leaving open only pharmacies and supermarkets and enforcing strict rules. In my home in Vancouver, with hundreds more confirmed and unconfirmed cases, kids are still attending day care, restaurants are still delivering food, domestic travel continues, and social isolation measures are not particularly enforced. Prince Edward Island, in contrast, has shut down daycares and even liquor stores; essential services that remain open in Ontario are different from those in Alberta. There is no one coordinated policy (as of yet) beyond the management of Canada’s borders. The ability to act fast and enforce has been part of NZ’s political history, and can be seen time and time again when it comes to social, economic, and educational policy. Canada as a large fragmented federal state has tended to act much more slowly and with much less coordination. In Canada, the way the country was formed, with power devolved largely to the provinces and territories, means there are 13 different actors who decide upon how the educational institutions are run and the priorities for adult training and education.

While these are unprecedented times, I can’t help but reflect on the fact that NZ is responding, policy-wise, to this pandemic in a way that reflects its history and national character, which is evidenced too in its adult education policy. The country’s 4‑level Covid-19 alert system clearly articulates the health and social measures to be taken from the “Prepare” stage (Level 1), through “Reduce” (Level 2), “Restrict” (Level 3), and, finally, “Eliminate” (Level 4, where the country is currently at).^7^ Similarly, New Zealanders have become very used to clearly developed and communicated levels of training and education, as articulated in and through the NZQA.^8^ As we have explored, with increasing accountability came increasing measurement and categorisation. Further, NZ is known as an incubator of social policy ideas across the world (such as, for example, Outcomes-Based Education, see, e.g., Martens & Starke, 2008); even now, within 24-hours of the lockdown, many Australians are looking East for a model to emulate (see., e.g., Alcorn 2020). There is a unity in the country that does not exist in places like Canada: there is one Indigenous people (Maori) who, while existing across different *iwi*, still speak one common language. This compares with over 50 First Nations in Canada with over 50 languages (sometimes vastly different), and numerous different cultural practices and histories (Government of Canada 2017). Equally important here are NZ’s geographic isolation as an island nation where it takes over 24 h to fly to Europe, its national pride of fairness and pragmatism, and the persistence of a tall poppy syndrome where no-one is to receive special treatment or blow their own trumpet (see., e.g., Wilson 2019). Whether getting the country on board with a highly coordinated, centralised, standardised response to education and training or with a highly coordinated, centralised, standardised response to the pandemic, government success has been remarkable.

## Concluding Remarks

Adult education systems across OECD countries vary vastly, not only between CMEs and LMEs, but also within them, which may or may not affect participation and outcomes. It is worth nothing that New Zealand was singled out in the latest GRALE report as having made major gains in employer-supported and other participation in adult education over the past years; it is also named as one of a few countries which has managed to admirably include vulnerable populations in adult learning and education (UIL 2019, p. 160). While Canada also has relatively high levels of participation and educational outcomes, it struggles in ways NZ does not given the long-arm of the NZQA. For example, in Canada foreign credential recognition is still a real challenge, as is cross-provincial labour mobility (Annen 2019). Further, by centralising and professionalising the field of adult education, whether in workplaces or community, NZ brought respect to the field (Walker 2011). Centralising and streamlining everything under the TEC provides coherence, consistency, and order to all non-compulsory education in New Zealand. At the same time, freedom, creativity, and possibilities for a more democratic and critical adult education may be lost.

The past has made the present. Inclusive liberal governments like Canada’s or NZ’s over the first two decades of the 2000s, have built on, tweaked, extended, repurposed, refined policies and structures built before them. Canada’s political structure, geography, history, and a host of other factors, meant that the institutions and structures of adult education were either never created or built on sand. Furthermore, proclamations of neoliberalism’s death have been uttered with increasing frequency and intensity since the global economic crisis (see, e.g., Jacques 2016). Nonetheless, while the planned laissez-faire utopian project (Polanyi 1957) may be over, neoliberalism’s spectre remains.

## Footnotes

1 Aotearoa is the Māori name for NZ, translated to “land of the long white cloud.” It is now common to refer to the country as Aotearoa New Zealand (abbreviated as NZ in this paper).

2 Pākehā is commonly used to refer to New Zealanders of European descent.

3 It’s interesting to note that NZ’s net migration rate has been almost double in recent years: 11.4 per 1000 compared to Canada’s 6.6/1000. See https://www.stats.govt.nz/news/new-zealand-net-migration-rate-remains-high and https://www.bbc.com/news/50061529.

4 Both countries’ gini coefficients are estimated at around 0.32 compared to Denmark’s 0.26. As a point of comparison, the US is at 0.39. See https://data.oecd.org/inequality/income-inequality.htm and http://archive.stats.govt.nz/browse_for_stats/snapshots-of-nz/nz-social-indicators/Home/Standard%20of%20living/income-inequality.aspx.

5 This is apparent in examining all recent TEC initiative found at https://www.tec.govt.nz/.

6 https://www.canada.ca/en/employment-social-development/campaigns/skills-boost.html.

7 See https://covid19.govt.nz/government-actions/covid-19-alert-system/.

8 See e.g., https://www.nzqa.govt.nz/studying-in-new-zealand/understand-nz-quals/.
